# Are complication rates lower with 4-Fr versus 6-Fr transfemoral arterial access – prospective audit at a single interventional radiology centre

**DOI:** 10.1186/s42155-018-0022-4

**Published:** 2018-08-23

**Authors:** Raymond Chung, Alex Weller, Robert Morgan, Anna-Maria Belli, Lakshmi Ratnam

**Affiliations:** 1 Diagnostic Radiology, Khoo Teck Puat Hospital, 90, Yishun Central, 768828 Singapore; 2 Radiology, Northwick Park Hospital, Watford Road, Harrow, HA1 3UJ UK; 3 Radiology, St. George’s University Hospitals NHS Foundation Trust, Blackshaw Road, London, SW17 0QT UK

**Keywords:** Access site related complications, Sheath size, Femoral arterial access

## Abstract

**Background:**

Femoral arterial access constitutes the first step in a significant proportion of interventional endovascular procedures. Whilst existing reports describe sheath size as an independent risk factor for bleeding complications in radial arterial access for coronary intervention, the influence of sheath size on overall complication rates and morbidity following femoral arterial access is not well described. This prospective single centre study reports our experience of vascular sheath size, patient and procedural factors in influencing complication rates following femoral arterial access. From April 2010 to May 2013, data was collected prospectively for all femoral arterial access procedures performed in the Interventional Radiology department of a tertiary hospital. For vascular sheath size <6-Fr, haemostasis was achieved by manual compression. For 6-Fr sheath size, a closure device was used in the absence of any contraindication.

**Results:**

Of the 320 femoral access cases with eligible inclusion criteria, 52.5% had 4-Fr whilst 47.5% had 6-Fr vascular sheaths inserted. Overall post procedure complications rates were significantly higher following 6-Fr sheath (17/152 (11.2%)) versus 4-Fr systems (3/168 (1.8%)) (p=0.0007) mostly comprising self-limiting hematoma. There was no significant difference in major complications that required escalation of treatment.

**Conclusion:**

No significant difference has been demonstrated between the use of either sheath systems for major complications. The practical limitations of a smaller system, combined with existing body of evidence, may not justify the routine use of 4-Fr sheath systems as the primary sheath size for all endovascular procedures.

## Background

Femoral arterial access constitutes the first step in a significant proportion of interventional endovascular procedures. Adequate training in safe arterial puncture (Fairley et al., 2016) and audit of outcomes are essential in minimising and recognising associated complications (Wagner et al., 2015; Rajebi & Rajebi, 2015). Complications predominantly relate to haemorrhage following arterial access. Existing reports describe sheath size as an independent risk factor for increased bleeding complications in radial arterial access for coronary intervention (Honda et al., 2012). Femoral bleeding complications have been shown to change with sheath size, duration of femoral arterial access, as well as intensity and duration of anticoagulation (Doyle et al., 2008; Cantor et al., 2007). However, the influence of sheath size on overall complication rates and morbidity following femoral arterial access is not well described and is of particular interest given the potential mitigation of these complications with arterial closure devices (Tzinieris et al., 2007; Das et al., 2011; Baumann et al., 2015).

There has been an increase in the range of equipment available, which are compatible with 4-French (Fr) sheaths, including angioplasty balloons and 0.018″ and 0.014″ systems. However, these are more expensive than their 6-Fr counterparts and the cost implications of using a smaller device and compatible systems is a consideration. Additionally, some technical limitations are associated with the use of the smaller systems. Patient and procedure related parameters also have an influence on complication rates. This study presents data from three audit cycles, conducted over three consecutive years, at a UK vascular IR (Interventional Radiology) centre. The aim was to assess the overall complication rates when using 4-French vascular access sheaths for femoral arterial endovascular interventions compared with 6-French sheaths; and evaluate the influence of patient and procedural parameters on these complication rates.

## Methods

A prospective single centre study was conducted at a tertiary teaching hospital IR unit, over three 6 month audit cycles and includes all vascular interventions performed via the transfemoral route in the interventional radiology suites between April 2010 and May 2013.

All transfemoral arterial access was performed using sterile technique and 1% lidocaine infiltration at the puncture site. Fluoroscopic and/or ultrasound anatomical landmarks over the femoral head/neck were used to ensure safe site of arterial access in the common femoral arterial (CFA) segment. The CFA was also located by ultrasound with reference to the inferior epigastric artery origin. In our institution, all inexperienced operators receive formal training in the basics of safe femoral arterial puncture, followed by close senior supervision, until they are competent in gaining safe femoral arterial access. Competence is assessed on the basis of 50 observed retrograde and 50 observed antegrade punctures. Wherever possible, sheaths were removed immediately post procedure in the interventional suite and 10 min of manual compression was applied for haemostasis. Following the use of a 6-Fr vascular access sheath, a closure device is deployed in the absence of any contraindications.

### Patient demographics

During the period of study, 392 arterial access procedures were performed, with 10 patients having two access sites. Fifty-eight cases were excluded as femoral sheath size was neither 4 Fr. nor 6 Fr. Additionally, nine 4-Fr and five 6-Fr cases were excluded as these were for alternative (non-femoral) access sites. In the remaining 320 procedures, 7 patients had bilateral femoral vascular access (4 patients having bilateral 4-Fr access, 2 patients having bilateral 6-Fr access, and 1 patient having one 4-Fr and one 6-Fr access). Patient clinical characteristics in the respective 4-Fr and 6-Fr subpopulation are presented in Table 1 (1 patient with bilateral access with 4 and 6-Fr systems was included in each demographic as an individual entity). Of the 314 patients, 186 were male and 128 were female. Mean age was 65.7 (range 21–94) years (unavailable in 10 patients). 41.2% had hypertension whilst 31.0% had diabetes. 45.3% were receiving anticoagulation prior to the procedure, consisting of 4 patients on warfarin, 43 on aspirin, 8 on clopidogrel, 7 on treatment dose low molecular weight heparin, 42 on prophylactic dose low molecular weight heparin, and 36 patients on > 1 of the aforementioned anticoagulation regimes. 2.3% had an INR > 1.5.

**Table 1:** Patient demographics

	4-Fr vascular sheath size population	6-Fr vascular sheath size population
No. of procedures	168	152
No. of patients	164	150
Age – mean (range) years	61.7 (21–92)	69.7 (21–94)
Sex M:F	71:93	115:35
Hypertension	52 (32.7%) – 5 unavailable	74 (50.3%) – 3 unavailable
Diabetes	47 (28.8%) – 1 unavailable	49 (33.3%) – 3 unavailable
Anticoagulation	3 unavailable	2 unavailable
Nil	93 (57.8%)	76 (51.4%)
Warfarin	3 (1.9%)	1 (0.7%)
Aspirin	25 (15.5%)	18 (12.2%)
Clopidogrel	5 (3.1%)	3 (2%)
Treatment dose heparin	3 (1.9%)	4 (2.7%)
Prophylactic dose low molecular weight heparin	19 (11.8%)	23 (15.5%)
Multiple	13 (8.1%)	23 (15.5%)
INR	1 unavailable	2 unavailable
< 1.5	160 (98.1%)	144 (97.3%)
> 1.5	3 (1.9%)	4 (2.7%)
Platelets (10^9^/L)	1 unavailable	3 unavailable
< 50	1 (0.6%)	1 (0.7%)
> 50, < 100	4 (2.5%)	4 (2.7%)
> 100	158 (96.9%)	142 (96.6%)

Procedure related complications were recorded for all patients operated on during the audit periods, with clinical data collated on an audit pro-forma, both immediately following each procedure and prior to discharge from hospital. Parameters relating to both the patient and the procedure were recorded, including: patient co-morbidities; body habitus; vessel calcification; haematological clotting status; concomitant anti-coagulation; operator experience; sheath size and the number of needle passes attempted to gain successful arterial access. The association of these factors, especially sheath size, with post-procedural complications was evaluated.

Complications were classified based on the Society of Interventional Radiology (SIR) guidelines. Minor complications are defined as those requiring no treatment. Major complications are those requiring treatment and further hospitalization, those requiring an unplanned increase in level of care, those with permanent adverse sequelae, and those resulting in death (Omary et al., 2003).

### Statistical analysis

Categorical variables were compared using Chi-squared analysis or Fisher’s exact test (SPSS software) and were adjusted for confounding factors. Statistical significance was defined as *P* < 0.05).

## Results

### Arterial access site characteristics

The arterial access site characteristics, operator seniority and methods of closure are recorded in Table 2. 75.6% were retrograde access, with the majority (95.3%) constituting puncture of the common femoral artery. 85% were successful with a single anterior wall puncture. 4-Fr vascular access sheaths were used in 52.5%, whilst 6-Fr sheaths were used in 47.5%. The vessels were calcified in 35.9% and the groins were scarred due to a previous surgical intervention in 16.6%.

**Table 2:** Arterial access characteristics/technique

	4-French (168 procedures)	6-French (152 procedures)	Access Site Characteristic for BOTH 4Fr and 6Fr procedures	
			Difference in complication rates (Cx = complication)	*p* - value
Patient side of puncture				
Left	72	69	Left (*n* = 141, Cx in 5.7%) vs right groin (*n* = 178, Cx in 6.7%)	*p* = 0.696
Right	95	83		
Unrecorded	1			
Seniority of operator				
Junior radiology trainee	16	16	Junior (*n* = 32, Cx in 0.0%) vs senior operator (*n* = 287, Cx in 7.0%) (fellow or consultant)	*p* = 0.240
Interventional radiology fellow	132	116		
Consultant	20	19		
Unrecorded	0	1		
Direction of puncture				
Retrograde	118	124	Retrograde (*n* = 242, Cx in 4.6%) vs antegrade (*n* = 78, Cx in 11.5%) puncture	*p = 0.027*
Antegrade	50	28		
Artery punctured				
External Iliac Artery	1	1		
CFA	161	144	CFA (*n* = 305, Cx in 6.2%) vs SFA (*n* = 13, Cx in 7.1%)	*p* = 0.604
SFA	6	7		
Number of passes				
1	142	130	Single (*n* = 272, Cx in 5.5%) vs multiple (*n* = 48, Cx in 10.4%) passes	*p* = 0.200
> 1	26	22		
Vessel calcification: Nil	43:125	72:80	Calcified (*n* = 115, Cx in 8.7%) vs non-calcified (*n* = 205, Cx in 4.9%)	*p* = 0.176
Scarred groin: Nil	14:154	39:113	Scarred (*n* = 53, Cx in 5.7%) vs non scarred (*n* = 267, Cx in 6.4%)	*p* > 0.999
Habitus				
Raised BMI: normal/low	57:111	37:114	Raised (*n* = 94, Cx in 5.3%) vs normal BMI (*n* = 225, Cx in 6.7%)	*p* = 0.651
Unrecorded		1		
Puncture technique				
Manual palpation	45	6	Manual palpation (*n* = 51, Cx in 2.0%) vs image guided (*n* = 269, Cx in 7.1%)	*p* = 0.219
Fluoroscopic guidance	9	7		
Ultrasound	62	81		
Combination	52	58		
Anticoagulation during procedure	2 unavailable	4 unavailable		
Nil	104	46	Anticoagulation (*n* = 164, Cx in 7.9%) vs none (*n* = 150, Cx in 3.3%)	*p* = 0.093
Intra-arterial heparin (3–5000 Units)	62	102		
Closure method				
Manual compression	163	87	Manual compression (*n* = 250, Cx in 6.4%) vs closure / compression device (*n* = 68, Cx in 5.8%)	*p* = > 0.999
Angioseal	4	54		
Exoseal	1	5		
Additional femstop	0	3		
Other (unrecorded) vascular closure device	0	1		
Vascular sheath left in-situ	0	1		
Unrecorded		1		

Arterial puncture was achieved with either ultrasound guidance alone (44.7%) or a combination of ultrasound and fluoroscopic guidance (34.4%); only 15.9% were accessed by manual palpation alone. 78.1% of access sites were sealed by manual compression. Punctures performed under manual palpation alone showed no significant difference in complication rates (1/51 patients = 2.0%) compared with those performed under image guidance (19/269 patients = 7.1%) (*p* = 0.219, Table 2).

### Access-site complications

**Fig. 1:**
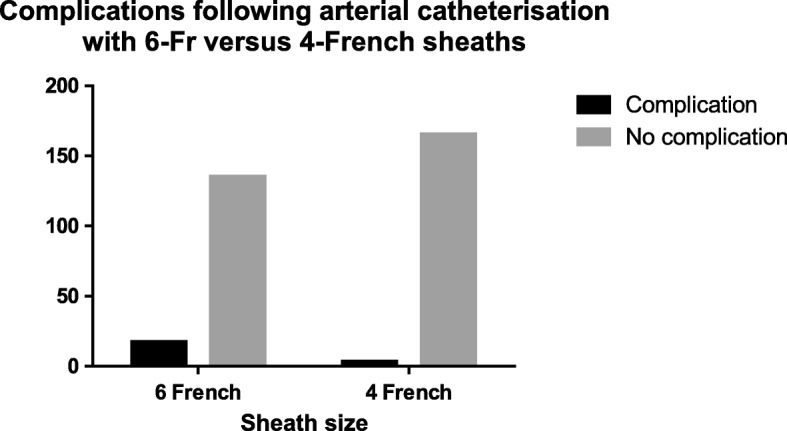
Bar-chart demonstrating lower complication rates following 4-Fr arterial catheterisation compared with 6-Fr systems. Legend: Complications following arterial catheterisation with 6-Fr versus 4-Fr sheaths

There were 20 complications in total (Table 3). The total number of patients having either 4-Fr or 6-Fr sheaths was 334, of which 320 were for femoral arterial access. The overall post procedure complication rates were significantly greater following catheterisation with 6-Fr sheaths (17/152 (11.2%)) versus 4-Fr systems (3/168 (1.8%)) (*p* = 0.0007) (Fig. 1). Compared with 4-Fr arterial catheterisation, the odds ratio (OR) for complication when using a 6-Fr system was 6.9 (95% CI 2.0–24.0). Of the 17 complications in the 6-Fr group; 1 haematoma required surgical management, 1 case of distal embolization required further endovascular management by Interventional Radiology and 1 case of distal embolization was managed medically. The remaining were self-limiting haematomas.

**Table 3:** Summary of complications between 4-Fr vs 6-Fr systems

	4 French		6 French	
	Self-limiting	Further active management	Self-limiting	Further active management
Complication				
Haematoma	1 antegrade	1 antegrade (surgery)	14 (8 retrograde, 6 antegrade)	1 antegrade (surgery)
Pseudoaneurysm		1 retrograde		
Distal embolism	–	–		1 retrograde (medical) 1 retrograde (endovascular by IR)

There were 3 complications in the 4-Fr group consisting of 1 self-limiting haematoma, 1 pseudoaneurysm formation, and 1 haematoma requiring surgical management. There was no significant difference between 4-Fr vs 6-Fr systems for major complications (defined as those delaying discharge or requiring escalation of treatment).

**Fig. 2:**
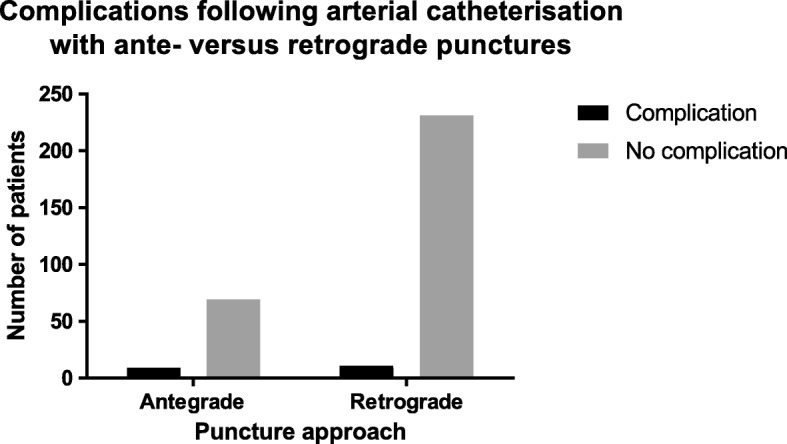
Bar-chart demonstrating lower complication rates following retrograde arterial catheterisation compared with the antegrade approach. Legend: Complications following arterial catheterisation with ante-versus retrograde punctures

Overall in both groups (4Fr and 6Fr), antegrade punctures (9/78 = 11.5%) were associated with significantly greater complication rates than retrograde punctures ((11/242 = 4.6%) (*p* = 0.027) (Fig. 2). Compared with the retrograde approach, the odds ratio for complication following antegrade puncture was 2.74 (95% CI 1.09–6.88). Considering *6Fr catheterisation alone*, complications were seen following 7/28 (25%) antegrade procedures and 10/124 (8.1%) retrograde procedures, comprising a significantly elevated risk for the former (*p* = 0.018). The OR for complication following a 6Fr antegrade procedure, compared with retrograde, was 3.8 (95% CI 1.3 to 11).

Only 0.9% of patients with complications required surgical intervention post procedure to correct access site complications. However; a comparison of complication rates between the remaining patient and procedure related parameters, including the seniority of the operator and the number of arterial passes, did not reach statistical significance.

In terms of anticoagulation, although 13/164 (7.9%) of patients receiving intra-procedural heparin developed complications, compared with 5/150 (3.3%) for those receiving no anticoagulation during the procedure, the difference between these two group did not reach significance (*p* = 0.093, Table 2). In addition, none of the 16 patients with pre-procedural INR > 1.5 or platelets < 100 developed complications. These data were well recorded. Specifically looking at complications in patients receiving anticoagulation *prior* to the procedure (warfarin, aspirin, clopidogrel, treatment dose heparin and prophylactic dose heparin), although there was a higher number of complications in the anticoagulation group compared with the ‘no anticoagulation’ group, no statistical difference was demonstrated (*p* = 0.115); 12/144 (8.3%) of those receiving prior anticoagulation developed complications, compared with 7/171 (4.1%), for those that had not (OR 2.13; 95% CI 0.816 to 5.562).

## Discussion

Access site bleeding complications have been reported in up to 19.3% of patients following femoral arterial catheterisation (Cantor et al., 2007), with major femoral bleeding associated with adverse outcomes, including prolonged hospital stay, increased blood transfusion requirements and decreased long-term survival (Doyle et al., 2008).

With the advent of low-profile devices, smaller sheaths have been introduced and intuitively, due to their smaller size, 4-Fr vascular access sheaths are expected to reduce the risk of post-procedural complications. The use of 4Fr access sheaths is associated with some technical difficulties such as a reduction in the ability to perform angiography via the sheath with a 4-Fr catheter in situ and increased difficulty with advancing some catheters within the sheath due to less physical space within the sheath. However, these difficulties are offset if there is reduction in complications. In a randomised study of patients undergoing diagnostic coronary angiography via the femoral arterial approach, 91 patients receiving 4-Fr arterial catheterisation were compared with 86 for whom 6-Fr catheters were used (Cantor et al., 2007). Complications were recorded in 22% of patients treated with 6-Fr catheters compared to 10% in those with 4-Fr catheters (*p* = 0.11); the majority of these complications were minor. Our results demonstrate a similar trend. Our data are also comparable to that of Durst et al. (Durst et al., 2007), confirming reduced rates of overall complications following femoral arterial access with 4-Fr compared to 6-Fr systems of 1.8% and 11.2%, respectively. With either system, however, the majority of complications were minor and the overall number of immediate access site related complications is low, with an even smaller number of major complications.

Previously reported independent risk factors for haemorrhagic complications following femoral arterial catheterisation include increased patient age, increasing sheath size, anticoagulation and raised body mass index (BMI) (Doyle et al., 2008; Wheatley et al., 2011). Our data confirm a higher complication risk with increasing sheath size (6-Fr compared with 4-Fr systems). In addition, we have demonstrated a higher rate of minor complications, when using an antegrade approach compared with a retrograde approach. Reasons for this may include difficulty in obtaining adequate manual compression in patients with high BMI, high skin entry sites for antegrade punctures, as well as initial guidewire entry into the profunda rather than superficial femoral artery resulting in prolonged manipulation and increase of minor groin hematoma as a consequence. Recent reports describe no added morbidity in planned antegrade superficial femoral arterial (SFA) punctures versus CFA punctures (Kweon et al., 2012). A further retrospective study comparing complications in antegrade punctures of the CFA (*n* = 50) vs SFA punctures (*n* = 130) using vascular closure systems similarly reported an overall low access site complication rate of 8.9%, with no significant difference between CFA and SFA access, or dependence on sheath size (Gutzeit et al., 2012).

No significant increase in complication rate was demonstrated in patients receiving anticoagulation prior to the procedure.

### Limitations

Despite the data being collated prospectively, the patients were not randomised. Furthermore, choice of vascular sheath size was reliant on the choice of the principal operator and therefore results may reflect an additional selection bias. Patients switching from 4-Fr to 6-Fr systems were also not recorded as a separate cohort, a subgroup of patients that were potentially liable to an increased complication risk from changing vascular access sheaths during a procedure. The number of major complications is low and for this subgroup of complications, a significant difference between the two catheterisation systems is not confirmed. A study involving a much larger number of patients would be required to conclusively state that there are no significant differences in major complication rates between 4-Fr and 6-Fr systems.

## Conclusion

Although our study has confirmed a significant reduction in overall complications when using a 4-Fr vascular sheath rather than a 6-Fr sheath in transfemoral arterial access for vascular interventional procedures, the majority of complications are minor, with no need for further intervention. The only additional factor demonstrating significance in complications was antegrade puncture. No significant difference has been demonstrated between the use of either sheath systems for major complications. Given the practical limitations of using a smaller system, these and earlier results do not justify the routine use of 4-Fr sheath systems as the primary sheath size for all endovascular procedures. As with all aspects of endovascular intervention, the choice of sheath size must be dictated by patient characteristics and the specific procedure planned, in order to balance safety, successful outcome and treatment economy.

## Abbrevations

BMI: Body mass index
CFA: Common femoral arterial
Cx: Complication
Fr: French
IR: Interventional Radiology
OR: Odds ratio
SFA: Superficial femoral arterial
SIR: Society of Interventional Radiology

## Authors’ information

Raymond CHUNG; rchung@doctors.org.uk, Consultant Radiologist, Department of Diagnostic Radiology, Khoo Teck Puat Hospital, Singapore.

Alex WELLER; alweller@gmail.com, Consultant Radiologist, Radiology Department, Northwick Park Hospital, UK.

Robert MORGAN; Robert.Morgan@stgeorges.nhs.uk, Consultant Radiologist, Radiology Department, St. George’s University Hospitals NHS Foundation Trust, UK.

Anna-Maria BELLI; Anna.Belli@stgeorges.nhs.uk, Professor of Interventional Radiology & Consultant Radiologist, Radiology Department, St. George’s University Hospitals NHS Foundation Trust, UK.

Lakshmi RATNAM; Lakshmi.Ratnam@stgeorges.nhs.uk, Consultant Radiologist, Radiology Department, St. George’s University Hospitals NHS Foundation Trust, UK.

## References

[CR1] Baumann F, Yates T, Gandhi R, Benenati J, Pena C, Katzen BT (2015). Single-center experience comparing the application of small-caliber versus large-caliber arterial access closure in a consecutive series of patients. J Vasc Interv Radiol.

[CR2] Cantor WJ, Mahaffey KW, Huang Z, Das P, Gulba DC, Glezer S (2007). Bleeding complications in patients with acute coronary syndrome undergoing early invasive management can be reduced with radial access, smaller sheath sizes, and timely sheath removal. Catheter Cardiovasc Interv.

[CR3] Das R, Ahmed K, Athanasiou T, Morgan RA, Belli AM (2011). Arterial closure devices versus manual compression for femoral haemostasis in interventional radiological procedures: a systematic review and meta-analysis. Cardiovasc Intervent Radiol.

[CR4] Doyle BJ, Ting HH, Bell MR, Lennon RJ, Mathew V, Singh M (2008). Major femoral bleeding complications after percutaneous coronary intervention: incidence, predictors, and impact on long-term survival among 17,901 patients treated at the Mayo Clinic from 1994 to 2005. JACC Cardiovasc Interv.

[CR5] Durst R, Lotan C, Nassar H, Gotsman M, Mor E, Varshitzki B (2007). Comparison of 4 and 6 French catheters for coronary angiography: real-world modeling. Isr Med Assoc J.

[CR6] Fairley SL, Lucking AJ, McEntegart M, Shaukat A, Smith D, Chase A et al (2016) Routine Use of Fluoroscopic-Guided Femoral Arterial Puncture to Minimise Vascular Complication Rates in CTO Intervention: Multi-centre UK experience. Heart Lung Circ 25(12):1203–1209 10.1016/j.hlc.2016.04.006 27265645

[CR7] Gutzeit A, van Schie B, Schoch E, Hergan K, Graf N, Binkert CA (2012). Feasibility and safety of vascular closure devices in an antegrade approach to either the common femoral artery or the superficial femoral artery. Cardiovasc Intervent Radiol.

[CR8] Honda T, Fujimoto K, Miyao Y, Koga H, Hirata Y (2012). Access site-related complications after transradial catheterization can be reduced with smaller sheath size and statins. Cardiovasc Interv Ther.

[CR9] Kweon M, Bhamidipaty V, Holden A, Hill AA (2012). Antegrade superficial femoral artery versus common femoral artery punctures for infrainguinal occlusive disease. J Vasc Interv Radiol.

[CR10] Omary RA, Bettmann MA, Cardella JF, Bakal CW, Schwartzberg MS, Sacks D (2003). Quality improvement guidelines for the reporting and archiving of interventional radiology procedures. J Vasc Interv Radiol.

[CR11] Rajebi H, Rajebi MR (2015). Optimizing common femoral artery access. Tech Vasc Interv Radiol.

[CR12] Tzinieris IN, Papaioannou GI, Dragomanovits SI, Deliargyris EN (2007). Minimizing femoral access complications in patients undergoing percutaneous coronary interventions: a proposed strategy of bony landmark guided femoral access, routine access site angiography and appropriate use of closure devices. Hell J Cardiol.

[CR13] Wagner J, Gandhi RT, Powell A (2015). Technical approach to Antegrade femoral access. Tech Vasc Interv Radiol.

[CR14] Wheatley BJ, Mansour MA, Grossman PM, Munir K, Cali RF, Gorsuch JM (2011). Complication rates for percutaneous lower extremity arterial antegrade access. Arch Surg.

